# ESTIMATION OF WORKING LIFE EXPECTANCY: APPLICATION OF A MULTISTATE MARKOV MODEL

**DOI:** 10.1093/geroni/igad104.1046

**Published:** 2023-12-21

**Authors:** Holendro Singh Chungkham

**Affiliations:** Stress Research Institute, Stockholm University, Stockholm, Stockholms Lan, Sweden

## Abstract

Longer life expectancy in Western countries has led to concerns about sustainable workforces amid the rising costs of securing aging populations. Retirement age is no more a good indicator of the end of the working life, because many older workers move in-and-out of workforce. A more useful measure is working life expectancy (WLE), which is the expected number of remaining years from a given age that a person will work. This measure considers transitions across different employment states. The method adopted for estimation of WLE is along the same line to estimate health expectancy (HE) based on cross-sectional data without considering transitions in different states of work over time. A better approach is based on multi-state survival models considering transitions among states over time using longitudinal data. These models assume a Markov Process to estimate transition probabilities. WLE is the weighted sum of duration of stay in a state weighted by the transition probabilities. The method adopted in IDEAR consortium is a multistate survival model assuming a first-order Markov process in continuous time to estimate transition probabilities. Transition probabilities are estimated by the msm package. This model takes care of interval censoring where the exact dates of transitions from one state to another is not known. WLEs are estimated by using the package elect (van den Hout, 2019) incorporating age as a time dependent variable by using a Gompertz model instead of the exponential model. The method is demonstrated to estimate WLE using Swedish Longitudinal Occupational Survey of Health (SLOSH).

